# Effects of patch contrast and arrangement on benefits of clonal integration in a rhizomatous clonal plant

**DOI:** 10.1038/srep35459

**Published:** 2016-10-19

**Authors:** Yong-Jian Wang, Xue-Ping Shi, Xiao-Jing Wu, Xue-Feng Meng, Peng-Cheng Wang, Zhi-Xiang Zhou, Fang-Li Luo, Fei-Hai Yu

**Affiliations:** 1 College of Horticulture & Forestry Sciences, Huazhong Agricultural University, Wuhan 430070, China; 2 School of Nature Conservation, Beijing Forestry University, Beijing 100083, China

## Abstract

The availabilities of light and soil water resources usually spatially co-vary in natural habitats, and the spatial pattern of such co-variation may affect the benefits of physiological integration between connected ramets of clonal plants. In a greenhouse experiment, we grew connected or disconnected ramet pairs [consisting of a proximal (relatively old) and a distal (relative young) ramet] of a rhizomatous herb *Iris japonica* in four heterogeneous environments differing in patch arrangement (reciprocal vs. parallel patchiness of light and soil water) and patch contrast (high vs. low contrast of light and water). Biomass of the proximal part, distal part and clonal fragment of *I. japonica* were all significantly greater in the intact than in the severed treatment, in the parallel than in the reciprocal patchiness treatment and in the high than in the low contrast treatment, but the effect of severing the connection between ramet pairs did not depend on patch arrangement or contrast. Severing the connection decreased number of ramets of the distal part and the clonal fragment in the parallel patchiness arrangement, but not in the reciprocal patchiness arrangement. Therefore, the spatial arrangement of resource patches can alter the effects of clonal integration on asexual reproduction in *I. japonica*.

Light, soil water and nutrients are commonly patchily distributed in nature^1^,^2^. Many clonal plants can spread horizontally and produce connected ramets located in microhabitats differing in resource supply^3^,^4^,^5^. Due to clonal integration, these connected ramets can exchange photosynthates, water and nutrients to improve the integrative performance of the whole clonal fragment^3^,^6^,^7^,^8^,^9^. However, the spatial pattern of resource patchiness varies greatly in nature, which may impact the benefits of clonal integration^10^,^11^,^12^.

The availabilities of light and soil water usually spatially co-vary in natural habitats^13^. In some habitats such as shrublands, high light intensity under sparse vegetation is associated with low soil water availability and low light intensity under dense vegetation is accompanied with high soil water availability^12^,^13^,^14^,^15^. In such environments with reciprocal patchiness of light and soil water, neither patch alone is ideal for plant growth^16^,^17^. In some other habitats such as fixed dunes, high light intensity is accompanied with high soil water availability and low light intensity with low soil water availability^18^,^19^. In such environments with parallel patchiness of light and soil water, patches with high light and high water are ideal for plant growth, whereas those with low light and low water are not^10^,^11^. Impacts of clonal integration depend strongly on the strength of the source-sink relationship between connected ramets that are located in different resource patches, and spatial patch arrangement (reciprocal vs. parallel) may affect the strength of the source-sink relationship. Therefore, the spatial pattern of patch arrangement may affect the benefit of clonal integration between connected ramets of clonal plants^10^,^11^.

Benefits of clonal integration may also depend on resource contrast between patches where connected ramets grow^4^,^8^,^20^. Contrast is a basic element of environmental heterogeneity^16^, and a minimal contrast between two patches is required to induce resource sharing (clonal integration) between connected ramets because clonal plants will sense the environment homogeneous if patch contrast is too low^8^,^21^. Thus, patch contrast can have a profound impact on benefits of clonal integration^7^,^8^,^22^,^23^. Previous studies have indeed shown that clonal integration benefited ramets in low resource patches more when resource contrast between patches was higher^7^,^8^,^20^,^23^. However, little is known about the interaction effect between patch contrast and patch arrangement on benefits of clonal integration.

We grew ramet pairs of a rhizomatous herb *Iris japonica* under four heterogeneous environments differing in spatial patch arrangement (reciprocal vs. parallel patchiness of light and soil water) and patch contrast (high vs. low contrast of light and water), with the rhizome connecting the two ramets of a pair either severed or left intact (to prevent or allow clonal integration). Specifically, we addressed the following questions: (1) Does rhizome connection (i.e. clonal integration) improve the growth of *I. japonica* in terms of biomass and ramet production under heterogeneous environments of both light and soil water? 2) Does spatial patch arrangement alter the impact of clonal integration on the growth of *I. japonica*? (3) Does spatial patch contrast alter the benefit of clonal integration on the growth of *I. japonica*? (4) Is there an interaction effect of patch arrangement and patch contrast on the benefit of clonal integration?

## Results

### Effects on the clonal fragment

**Figure 1:**
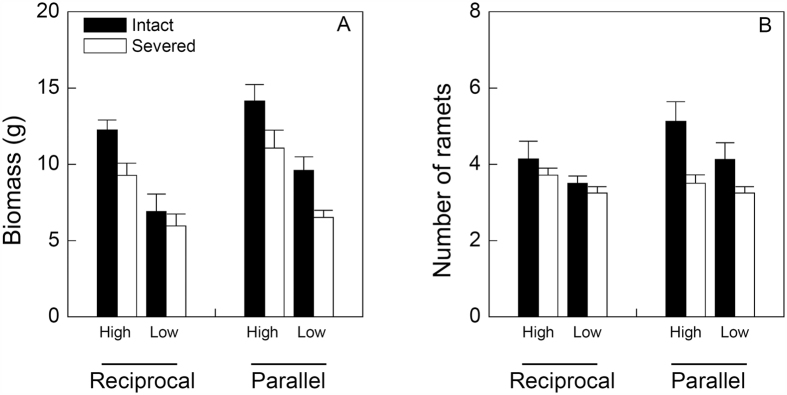
Effects of patch arrangement (reciprocal vs. parallel), patch contrast (high vs. low) and rhizome severing (intact vs. severed) on the growth of the whole clonal fragment (proximal plus distal part) of *I. japonica*. Bars and vertical lines are mean and SE.

Patch arrangement, patch contrast and rhizome severing significantly affected biomass of the clonal fragment of *I. japonica* (Table 1A). Biomass of the clonal fragment was significantly larger in the parallel than in the reciprocal arrangement, in the high than in the low contrast and in the intact than in the severed treatment (Fig. 1A). However, effects of rhizome severing on biomass of the clonal fragment did not depend significantly on patch arrangement or patch contrast (Table 1A, *P* > 0.05 for A × S and C × S). Number of ramets of the clonal fragment was also higher in the high than in the low contrast treatment and in the intact than in the severed treatment (Table 1A, Fig. 1B), but the negative effect of rhizome severing was more pronounced in the parallel than in the reciprocal treatment, as indicated by a significant effect of patch arrangement × rhizome severing (Table 1A, Fig. 1B). There was no significant three-way interaction effect of patch arrangement, patch contrast and rhizome severing on biomass or number of ramets of the clonal fragment (Table 1A).

### Effects on the proximal part

**Figure 2:**
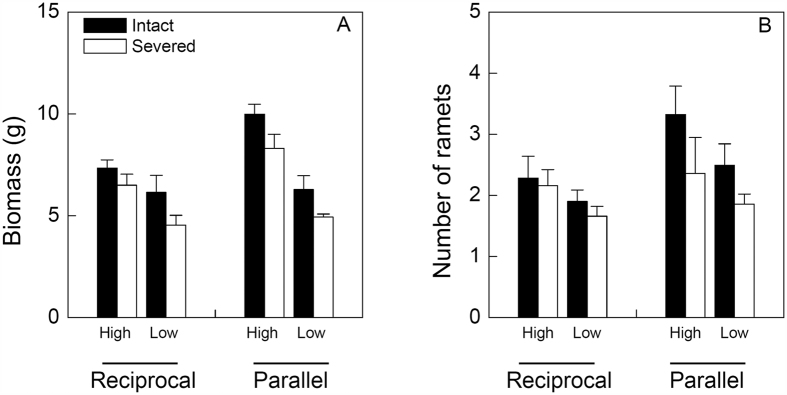
Effects of patch arrangement (reciprocal vs. parallel), patch contrast (high vs. low) and rhizome severing (intact vs. severed) on the growth of the proximal part of *I. japonica*. Bars and vertical lines are mean and SE.

Patch arrangement, patch contrast and rhizome severing significantly affected biomass of the proximal part of *I. japonica* (Table 1B). Biomass of the proximal part was significantly larger in the parallel than in the reciprocal arrangement, in the high than in the low contrast and in the intact than in the severed treatment (Fig. 2A). However, effects of rhizome severing on biomass of the proximal part did not depend on patch arrangement or patch contrast (Table 1B, *P* > 0.05 for A × S and C × S). Number of ramets of the proximal part was higher in the high than in the low contrast treatment, but was not significantly affected by patch arrangement or rhizome severing (Table 1B, Fig. 2B). There was no significant three-way interaction effect of patch arrangement, patch contrast and rhizome severing on biomass or number of ramets of the proximal part (Table 1B).

### Effects on the distal part

**Figure 3:**
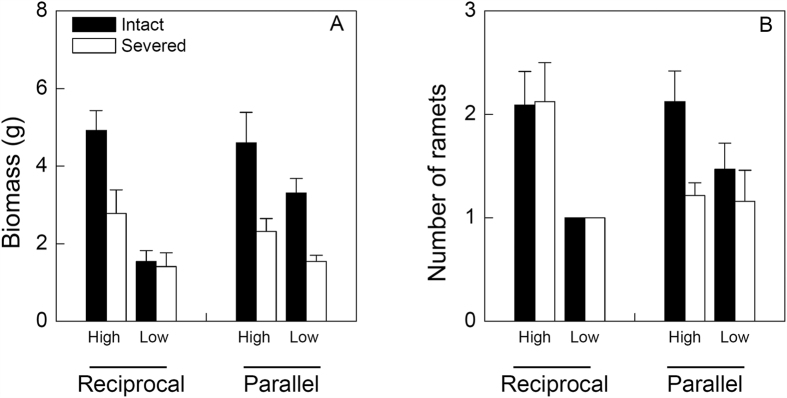
Effects of patch arrangement (reciprocal vs. parallel), patch contrast (high vs. low) and rhizome severing (intact vs. severed) on the growth of the distal part of *I. japonica*. Bars and vertical lines are mean and SE.

Biomass of the distal part was significantly larger in the parallel than in the reciprocal arrangement, in the high than in the low contrast and in the intact than in the severed treatment (Fig. 3A). However, effects of rhizome severing on biomass of the distal part did not depend on patch arrangement or patch contrast (Table 1C, *P* > 0.05 for A × S and C × S). Number of ramets of the distal part was higher in the high than in the low contrast treatment (Table 1C, Fig. 3B). In the reciprocal patch arrangement, rhizome severing had no effect on number of ramets of the distal part, but in the parallel patch arrangement rhizome severing decreased it, as indicated by a significant interaction effect of patch arrangement × rhizome severing (Table 1C; Fig. 3B). There was no significant three-way interaction effect of patch arrangement, patch contrast and rhizome severing on biomass or number of ramets of the distal part (Table 1C).

## Discussion

In heterogeneous environments, clonal integration significantly improved the growth of both the proximal and the distal part of *I. japonica*, thereby markedly increasing the growth of the whole clonal fragment. This result agrees with the finding of a previous meta-analysis demonstrating that clonal integration generally increases the growth of whole clonal fragments in heterogeneous environments^6^. While the effects of clonal integration on biomass did not depend on patch arrangement, its effects on number of ramets of both the distal part and the whole clonal fragment depended significantly on patch arrangement. These results suggest that the spatial arrangement of resource patches can alter the effects of clonal integration on asexual reproduction.

Clonal integration increased number of ramets of both the distal part and the whole clonal fragment of *I. japonica* in the parallel patch arrangement but had little impacts in the reciprocal patch arrangement. Under parallel patchiness, the gradients of both resources (light and water) were in the same direction so that the internal source-sink relationships of photosynthates and water between the proximal and the distal part were very likely to be in the same direction^10^,^11^,^17^,^24^. By contrast, under reciprocal patchiness, the gradients of light and water were in the opposite directions so that the the internal source-sink relationships of photosynthates and water between the proximal and the distal part were likely to be in the opposite directions^10^,^11^,^20^,^24^. Resource transportation may be more efficient when the source-sink resources of both photosynthates and water are in the same direction than when they are in the opposite directions. This increased efficiency of resource transportation may have contributed to the production of more but smaller ramets in the parallel patch arrangement.

The benefits of clonal integration on biomass or number of ramets of *I. japonica* did not depend significantly on patch contrast that was used in our experiment. However, Friedman & Alpert (1991) found that clonal integration increased growth of *Fragaria chiloensis* when patch contrast of light and nutrients was high, but not when patch contrast was low^20^. Similarly, effects of clonal integration on growth of *Diplopterygium glaucum* also depended on the degree of patch contrast and were significant only under relatively high patch contrast^22^. In our study, the possible reason for lack of an effect of patch contrast is that the resource contrast in the low patch contrast treatment already exceeded the minimum required by clonal integration of *I. japonica*, and that the resource contrast in the high patch contrast treatment was not large enough to change the effect of clonal integration.

Regardless of clonal integration, biomass of the proximal part, the distal part and the whole clonal fragment of *I. japonica* were significantly larger in the parallel than in the reciprocal arrangement. Previous study also showed that biomass of the whole clonal fragment of *F. orientalis* was larger in parallel patchiness than in reciprocal patchiness^11^. In the parallel arrangement, high light was always accompanied with high water, and medium light was always accompanied with medium water. Thus, disconnected ramets can grow much better in these resource-rich patches^24^,^25^,^26^,^27^,^28^. Furthermore, the high efficiency of resource transportation between connected ramets when the source-sink relationships of both photosynthates and water are in the same direction may have also promoted growth of *I. japonica*.

Growth of the whole clonal fragment of *I. japonica* was higher under the high than under the low patch contrast treatment. This is very likely because the overall resource levels of light and water for the whole clonal fragment differed between the two patch contrast treatments. In our experiment, the low contrast was between the medium and the low level of light or water, but the high contrast was between the high and the low level of light and water. As a result, the overall levels of light and water were higher in the the high contrast than in the low contrast treatment. The higher overall resource levels in the high patch contrast treatment may have contributed to the increased growth of the whole clonal fragment of *I. japonica.*

We conclude that the spatial arrangement of resource patches can alter the effects of clonal integration on asexual reproduction of *I. japonica*. Because the overall resource levels in the two patch contrast treatments were not the same, we cannot fully eliminate the confounding effect of resource levels from those of patch contrast. Thus, further studies testing effects of patch contrast should consider to set the overall resource levels for the whole clonal fragment the same across all the treatments. In our study, we considered only the effects of clonal integration during the vegetative growth period of *I. japonica*, and did not test those on sexual reproduction, i.e. the real fitness measures. Studies examining the effects on both asexual reproduction (clonal growth) and sexual reproduction (flowering and fruiting) are more valuable.

## Materials and Methods

### The species and material preparation

*Iris japonica* Thunb. (Iridaceae), a perennial clonal herb, is widely distributed in Asia^29^,^30^. This species produces long rhizomes with rooted ramets on its nodes. Most rhizomes are distributed in the topsoil of less than 5 cm deep^30^. The distance between adjacent ramets varies between 5 to 15 cm, and population density can be higher than 20 ramets m^−2^. Ramets of the same clone are often located in heterogeneous light and water environments from forest understories to open areas^12^,^30^. Without disturbance, rhizome connections between ramets can last up to one year in natural conditions. Disturbance such as animal trampling and rodent grazing can easily break rhizome connections. It flowers from March to April and fruits from May to June. Viable seeds are produced each year, but seedlings occur at very low densities in established populations. Clonal growth is the main method for its population spread^12^.

In early January 2014, more than 100 connected ramet pairs of *I. japonica* were collected from five locations in an evergreen broad-leaved forest on Shizi Mountain in Hubei Province, China. The distance between any two locations was at least 100 m to increase the chance of sampling different genets. The ramet pairs were cultivated in a greenhouse at Huazhong Agricultural University in Hubei Province, China. One ramet in each pair was recognized as the initial proximal part, indicating its relative proximity to the mother ramet, while the other as the initial distal part. After two weeks of cultivation, we selected 84 similar-sized ramet pairs of *I. japonica*, and each ramet of a pair had three leaves and some roots. Of them, 20 ramet pairs were randomly selected for measuring initial dry mass (mean ± SE: 0.842 ± 0.113 g for the proximal ramet and 0.733 ± 0.053 g for the distal ramet), and the other 64 pairs were used for the experiment described below.

### Experimental design

The experiment used a factorial design with two levels of rhizome severing (severed or not, i.e. prevent or allow clonal integration), two levels of patch contrast (high vs. low) and two levels of patch arrangement (parallel vs. reciprocal). In each treatment, the proximal and the distal ramet of a pair was planted in the two parts (each 25 cm long ×25 cm wide ×30 cm deep) of a plastic container (50 cm long ×25 cm wide ×30 cm deep), separated by a physical divider (25 cm long ×25 cm deep). The physical barrier was built in between the two patches of each container in the way that water flow between patches was prevented. A 2 × 2 cm slice was made on the barrier so that the rhizome connecting the two ramets of a pair could pass through.

**Figure 4:**
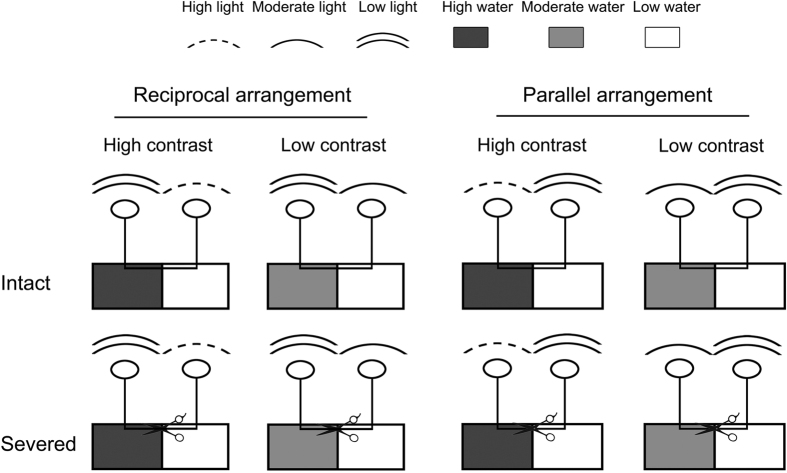
Experimental design. The experiment used a factorial design with two rhizome severing treatments (intact vs. severed), two patch contrast treatments (high vs. low) and two patch arrangement treatments (reciprocal vs. parallel). In the high contrast treatment, the proximal ramet of a pair was subjected to the high water and/or high light treatment, while the distal ramet was subjected to the low water and/or low light treatment; in the low contrast treatment, the proximal ramet was subjected to the moderate water and/or moderate light treatment, while the distal ramet was subjected to the low water and/or low light treatment. In the parallel arrangement, high light was always accompanied with high water, moderate light with moderate water and low light with low water; in the reciprocal arrangement, high and moderate light were accompanied with low water and low light with high water or moderate water.

The rhizome connecting the two ramets of a pair was cut off in the rhizome-severing treatment and left connected in the intact (not severed) treatment (Fig. 4). In the high contrast treatment, the proximal ramet of a pair was subjected to the high water and/or high light treatment, while the distal ramet was subjected to the low water and/or low light treatment. In the low contrast treatment, the proximal ramet was subjected to the moderate water and/or moderate light treatment, while the distal ramet was subjected to the low water and/or low light treatment (Fig. 4). In the parallel arrangement, high light was always accompanied with high water, moderate light with moderate water and low light with low water. In the reciprocal arrangement, high light and moderate light were accompanied with low water and high water and moderate water with low light (Fig. 4). There were eight replicates in each treatment.

Each container was filled with a mix of sand and yellow-brown soil (v/v: 1:1) and 15 g slow release fertilizer (Osmocote, N–P–K: 15–9–12, 5–6 month). All ramet pairs were allowed to recover for two weeks before the start of the experiment. High light was the natural light in the greenhouse, and moderate and low light were 55% and 10% of high light by covering the ramets with two types of neutral shading nets, respectively. The high, moderate and low water patches were created by adding 200, 110 and 20 mL water to the ramets every one to four days, respectively, depending on how fast the soil dried. Soil water content was monitored in four replicates of each treatment during the experimental period (by Soil Moisture Meter TZS-II, HEB Biotechnology Co., Xi’an, China). Ramet pairs were randomly assigned to positions on a bench in the glasshouse.

The experiment ran for four months, from 1 March to 1 July 2014. During the experiment, the mean temperature was 26.0 °C and the mean relative humidity was 70.5% (by Amprobe TR300, Amprobe, Everett, WA, USA). The light intensity in the greenhouse was about 85% of that outside.

### Measurements and analyses

We counted number of ramets in the proximal and the distal parts (patches) separately. Then, all ramets in each part were harvested, dried at 80 °C for 48 h and weighed. Biomass and number of ramets of a clonal fragment were the sum of those of the proximal and the distal part.

We used three-way ANOVAs to test effects of rhizome severing (severed vs. intact), patch arrangement (reciprocal vs. parallel), patch contrast (high vs. low) and their interactions on biomass and number of ramets of the clonal fragment, the proximal part and the distal part. All statistical analyses were carried out with SPSS 13.0 (SPSS, Chicago, IL, USA). Prior to ANOVAs, data of biomass was transformed to the square root to increase normality and homogeneity of variance. The differences were considered significant if *P* < 0.05.

## Additional Information

**How to cite this article**: Wang, Y.-J. *et al.* Effects of patch contrast and arrangement on benefits of clonal integration in a rhizomatous clonal plant. *Sci. Rep.* **6**, 35459; doi: 10.1038/srep35459 (2016).

## Figures and Tables

**Table 1:** Results of three-way ANOVAs for effects of patch arrangement (parallel vs. reciprocal patch), patch contrast (high vs. low) and rhizome severing (severed vs. intact) and their interactions on the growth of the whole clonal fragment (A), the proximal part (B) and the distal part (C) of Iris japonica.

	Arrangement (A)	Contrast (C)	Severing (S)	A × C	A × S	C × S	A × C × S
(A) Clonal fragment							
Biomass	7.11^**^	49.32^***^	16.53^***^	0.20^ns^	1.53^ns^	0.28^ns^	0.34^ns^
No. of ramets	2.70^ns^	5.84^*^	12.80^***^	0.01^ns^	4.44^*^	0.76^ns^	0.23^ns^
(B) Proximal part							
Biomass	21.29^***^	46.86^***^	10.02^**^	1.80^ns^	1.50^ns^	1.66^ns^	0.65^ns^
No. of ramets	3.21^ns^	5.03^*^	1.51	1.10^ns^	0.29^ns^	0.29^ns^	0.05^ns^
(C) Distal part							
Biomass	5.31^*^	12.05^***^	16.12^***^	0.34^ns^	1.67^ns^	2.89^ns^	0.82^ns^
No. of ramets	0.28^ns^	19.44^***^	6.63^**^	3.52^ns^	6.89^**^	0.69^ns^	0.47^ns^

The given are *F* values and significance levels (^***^P < 0.001, ^**^P < 0.01, ^*^P < 0.05 and ^ns^ *P* ≥ 0.05). Degree of freedoms were 1, 64 for all the effects.
